# Palmitoylethanolamide Promotes White-to-Beige Conversion and Metabolic Reprogramming of Adipocytes: Contribution of PPAR-α

**DOI:** 10.3390/pharmaceutics14020338

**Published:** 2022-01-31

**Authors:** Chiara Annunziata, Claudio Pirozzi, Adriano Lama, Martina Senzacqua, Federica Comella, Antonella Bordin, Anna Monnolo, Alessandra Pelagalli, Maria Carmela Ferrante, Maria Pina Mollica, Angelo Iossa, Elena De Falco, Giuseppina Mattace Raso, Saverio Cinti, Antonio Giordano, Rosaria Meli

**Affiliations:** 1 Department of Pharmacy, School of Medicine, University of Naples Federico II, 80131 Naples, Italy; chiara.annunziata@unina.it (C.A.); claudio.pirozzi@unina.it (C.P.); adriano.lama@unina.it (A.L.); federica.comella@unina.it (F.C.); mattace@unina.it (G.M.R.); 2 Department of Experimental and Clinical Medicine, Marche Polytechnic University, 60020 Ancona, Italy; m.senzacqua@staff.univpm.it (M.S.); s.cinti@univpm.it (S.C.); a.giordano@univpm.it (A.G.); 3 Department of Medical-Surgical Sciences and Biotechnologies, Faculty of Pharmacy and Medicine, Sapienza University of Rome, 04100 Latina, Italy; antonella.bordin@uniroma1.it (A.B.); angelo.iossa@uniroma1.it (A.I.); elena.defalco@uniroma1.it (E.D.F.); 4 Department of Veterinary Medicine and Animal Production, University of Naples Federico II, 80137 Naples, Italy; anna.monnolo@unina.it (A.M.); mariacarmela.ferrante@unina.it (M.C.F.); 5 Department of Advanced Biomedical Sciences, University of Naples Federico II, 80131 Naples, Italy; alpelaga@unina.it; 6 Institute of Biostructure and Bioimaging, National Research Council (CNR), 80134 Naples, Italy; 7 Department of Biology, University of Naples Federico II, 80126 Naples, Italy; mariapia.mollica@unina.it; 8 Mediterranea Cardiocenter, 80122 Naples, Italy

**Keywords:** brown and white adipose tissue, beige adipocytes, adipocyte remodeling, peroxisome proliferator-activated receptor-a, leptin signaling, human adipose stromal cells

## Abstract

The potential role of brown and beige adipose tissue against obesity has been recognized. Browning, or beiging of white adipose tissue (WAT) is associated with the remodeling of adipocytes and the improvement of their metabolic and secretory functions. Here, palmitoylethanolamide (PEA) restore the plasticity of brown and white adipocytes impaired in mice on a high-fat diet (HFD). Young male C57Bl/6J mice were fed with control (STD) diet or HFD for 12 weeks. Ultramicronized PEA (30 mg/kg/die p.o.) was administered for an additional 7 weeks, together with HFD. PEA recovered interscapular brown fat morphology and function, increasing UCP1 positivity, noradrenergic innervation, and inducing the mRNA transcription of several specialized thermogenic genes. PEA promotes the beige-conversion of the subcutaneous WAT, increasing thermogenic markers and restoring leptin signaling and tissue hormone sensitivity. The pivotal role of lipid-sensing peroxisome proliferator-activated receptor (PPAR)-α in PEA effects was determined in mature 3T3-L1. Moreover, PEA improved mitochondrial bioenergetics in mature adipocytes measured by a Seahorse analyzer and induced metabolic machinery via AMPK phosphorylation. All these outcomes were dampened by the receptor antagonist GW6471. Finally, PEA induced adipogenic differentiation and increased AMPK phosphorylation in human adipose-derived stromal cells (ASCs) obtained from subcutaneous WAT of normal-weight patients and patients with obesity. We identify PEA and PPAR-α activation as the main mechanism by which PEA can rewire energy-storing white into energy-consuming brown-like adipocytes via multiple and converging effects that restore WAT homeostasis and metabolic flexibility.

## 1. Introduction

In mammals, white (WAT) and brown (BAT) adipose tissues with opposing functions (lipid storage vs. thermogenesis) coexist into multiple visceral and subcutaneous fat depots. The adipose tissue is a convertible organ able to plastically adapt its metabolic, endocrine, and thermogenic functions to endogenous or exogenous stimuli [1]. Visceral and subcutaneous fat depots contain different cell types in stromal vascular fraction and extensively differ in immune cell infiltration, vascularization, and innervation [2]. BAT has a different developmental origin, progenitor cells, morphological appearance, and endocrine roles. Whereas WAT represents the main energy reservoir of the body, BAT is a highly specialized tissue that burns energy to produce heat providing non-shivering thermogenesis via the mitochondrial uncoupling protein (UCP)1 [3]. Some adipocyte progenitors and white adipocytes residing into WAT depots are capable of browning or beiging, by which thermogenic brown-like adipocytes emerge in response to distinctive stimuli [3]. Importantly, this phenotypic change of the adipose organ has proved to be effective in the protection against the metabolic disorders associated with obesity and diabetes. Thus, a better understanding of the molecular mechanisms at the basis of beiging or browning can be extremely useful to exploit new therapeutic strategies to counteract the increasing incidence of metabolic diseases [4,5]. The pharmacological research aims at identifying targets and molecules able to switch on the beige phenotype and to re-establish metabolic plasticity of the adipose organ. Among lipid-sensing nuclear receptors such as peroxisome proliferator-activated receptors (PPARs) [6], PPAR-α can be considered an attractive target. PPAR-α connects the nutritional inputs to the activation of specific cellular gene programs, that are involved in whole-body carbohydrate and lipid metabolism, increasing free fatty acid oxidation, improving insulin resistance and energy expenditure in obesity [7,8]. Obesity-induced metabolic alterations drive changes from healthy to dysfunctional adipocytes contributing to systemic chronic inflammation and insulin resistance and determining adipose tissue inability in adapting to exogenous stimuli [4]. A close interplay interestingly exists among adipocytes, sympathetic nervous and immune systems, and this crucial crosstalk is deeply affected when obesity occurs [9].

Palmitoylethanolamide (PEA) belongs to the bioactive lipid family of N-acylethanolamines (NAEs), and several rapid non-genomic and delayed genomic mechanisms of action have been identified [10,11,12]. A cohort study recently investigated the effect of dysmetabolism on the circulating levels of PEA and other congeners, whose concentrations and ratio were profoundly altered in obese patients [13]. Previous findings demonstrate that PEA can improve energy balance in a rat model of mild obesity [14], in diabetic rats [15], and in high-fat diet (HFD)-induced obesity in mice [12]. Interestingly, in liver, PEA can activate AMP-activated protein kinase (AMPK) [12], a cellular energy sensor also involved in the regulation of metabolic activity of BAT and WAT [16]. The modulation of AMPK by pharmacological agents is crucial for the maintenance of mitochondrial integrity in adipocytes, and the lack of adipocyte AMPK worsened insulin resistance and non-alcoholic fatty liver disease (NAFLD) through adipose tissue alteration [17]. Recently, we demonstrated that the obese phenotype of HFD-fed mice was improved by long-term PEA treatment restoring glucose and lipid homeostasis [12]. Moreover, we demonstrated that PEA recovered the alteration of serum leptin and adiponectin and re-established their physiological ratio. The altered adiponectin/leptin ratio is indicative of a dysfunctional adipose tissue [18], and highly correlated with markers of chronic low-grade “metabolic” inflammation, also called metainflammation [19,20]. Little is known about the adipose-hormonal adaptation during white-to-beige adipocyte conversion.

Here, PEA recovers adipose tissue function (BAT and WAT morphology and features) and increases thermogenic capability, restoring the leptin signaling pathway and tissue hormone sensitivity. These data provide compelling evidence for unknown crosstalk among PEA, adipocytes, inflammation, and the sympathetic nervous system, that can promote white-to-beige fat conversion. The role of PPAR-α-mediated metabolic reprogramming of adipocytes by PEA was determined in vitro on mature 3T3-L1. PEA effects were also evaluated in human adipose-derived stromal cells (ASCs) obtained from normal-weight patients (NWP) and patients with obesity (PwO).

## 2. Materials and Methods

### 2.1. Animals and Experimental Protocol

All procedures involving the animals were carried out according to Institutional Guidelines and complied with the Italian D.L. (No. 26 of 4 March 4 2014) of the Italian Ministry of Health (approved under protocol no. 982/2017-PR) and with European directive 2010/63/EU guidelines, including the ARRIVE guidelines 2.0. Male mice, 6-week-old C57Bl/6J (Charles River, Wilmington, MA, USA), were housed at an environmental temperature of 22 ± 1 °C with a 12:12 h light-dark cycle. First, the animals were randomly divided into 2 groups, considering a comparable body weight mean between groups: control group receiving standard diet (STD, Mucedola srl, Milan, Italy) and mice fed with a high-fat diet (HFD). After 12 weeks, when the obesity was full-blown, HFD mice were divided into 2 subgroups (n = 15 each group): HFD mice receiving vehicle and HFD receiving PEA p.o. (HFD + PEA). STD mice also received PEA vehicle p.o. by gavage (n = 15). Ultramicronized PEA (kindly provided by Epitech Group S.p.A., Padua, Italy) was suspended in carboxymethyl cellulose (1.5%) and administered at the dose of 30 mg/kg/die by oral gavage. The treatment lasted 7 weeks. Here, we used the PEA dose that previously showed peripheral and central metabolic efficacy in HFD-induced obese mice [11,12]. A more detailed description of the methods can be found in the Supporting Information (Appendix A).

### 2.2. Morphological Studies

Alternate sections of interscapular BAT (iBAT) and subcutaneous WAT (scWAT) were stained with hematoxylin and eosin (H & E) to assess morphology, and immunohistochemical procedures were carried out to evaluate tyrosine hydroxylase (TH) and UCP1 tissue protein expression. The morphological study methods are detailed in the Supporting information (Appendix A).

### 2.3. Real-Time Semi-Quantitative PCR

Total RNA of adipose tissue was obtained using RNeasy lipid tissue (Qiagen, Hilden, Germany). cDna was synthesized using a high-capacity cDNA reverse transcription kit (Thermo Fisher Scientific, Waltham, MA, USA). PCRs were performed with a Bio-Rad CFX96 Connect real-time PCR system instrument and software (Bio-Rad Laboratories srl, Milan, Italy), as previously reported [21]. Each cDNA sample (500 ng) was mixed with 2× QuantiTech SYBRGreen PCR Master Mix (Qiagen, Hilden, Germany,) and validated primers (Appendix A, Table A1). Data were normalized to Actb for BAT and Rn18S for scWAT as a housekeeping gene, and the data were analyzed according to 2^−ΔΔCt^ method.

### 2.4. Cell Culture and In Vitro Experiments

3T3-L1: 3T3-L1 mouse fibroblast cells were differentiated into adipocytes, as previously described [22]. Mature adipocytes were incubated with GW6471 (10 μM). One hour later, cells were stimulated with PEA (3 μM, dissolved in EtOH) and harvested after twenty-four hours. In a further set of experiments, a Mito stress test was performed as previously described [23], by a Seahorse XFe24 analyzer. A more detailed description of methods can be found in the Supporting Information (Appendix A). ASCs: Surgical procedures were conducted at “Sant’Andrea” Hospital, Rome. The study was approved by the Ethical Committee of the hospital (reference 49_2013/28.01.2016) [24]. Written informed consent was obtained from the patients, before starting all the surgical and laboratory procedures. The methodology described in this study has been conducted in compliance with the tenets of the Declaration of Helsinki for experiments involving human tissues. Medical records of each patient were collected in a clinical database (the prevalence of obesity is defined as body mass index ≥30, according to the World Health Organization criteria). All data were de-identified and analyzed anonymously. ASCs were isolated from subcutaneous fat depots of NWP and PwO (body mass index: 39.075, SD ± 7.36) as previously reported [25,26]. Further information on methods and treatments are included in Appendix A.

### 2.5. Western Blot

scWAT (about 100 mg) and cells were lysed in RIPA buffer (50 mM Tris·HCl, pH 7.4, 1% Triton X-100, 150 mM NaCl, and 1 mM EDTA) in the presence of protease inhibitors. Then, the samples were homogenized on ice for 15 min, and centrifuged twice at 14,000× *g* for 15 min, to separate the lipophilic phase and collect the aqueous intermediate phase. Further details about methods are included in the Supporting Information (Appendix A).

### 2.6. Statistical Analysis

A priori sample size was determined by a power calculation using G*power software, considering the body weight and glycemia as primary outcome. Data are presented as mean value ± SEM. Statistical analysis was performed by one-way ANOVA followed by Bonferroni’s post hoc test for multiple comparisons, for both in vivo and in vitro experiments. The analysis was performed using GraphPad Prism 8 (GraphPad Software, San Diego, CA, USA) with a level of significance of *p* < 0.05.

## 3. Results

### 3.1. PEA Recovered BAT Morphology and Function Altered by HFD

**Figure 1:**
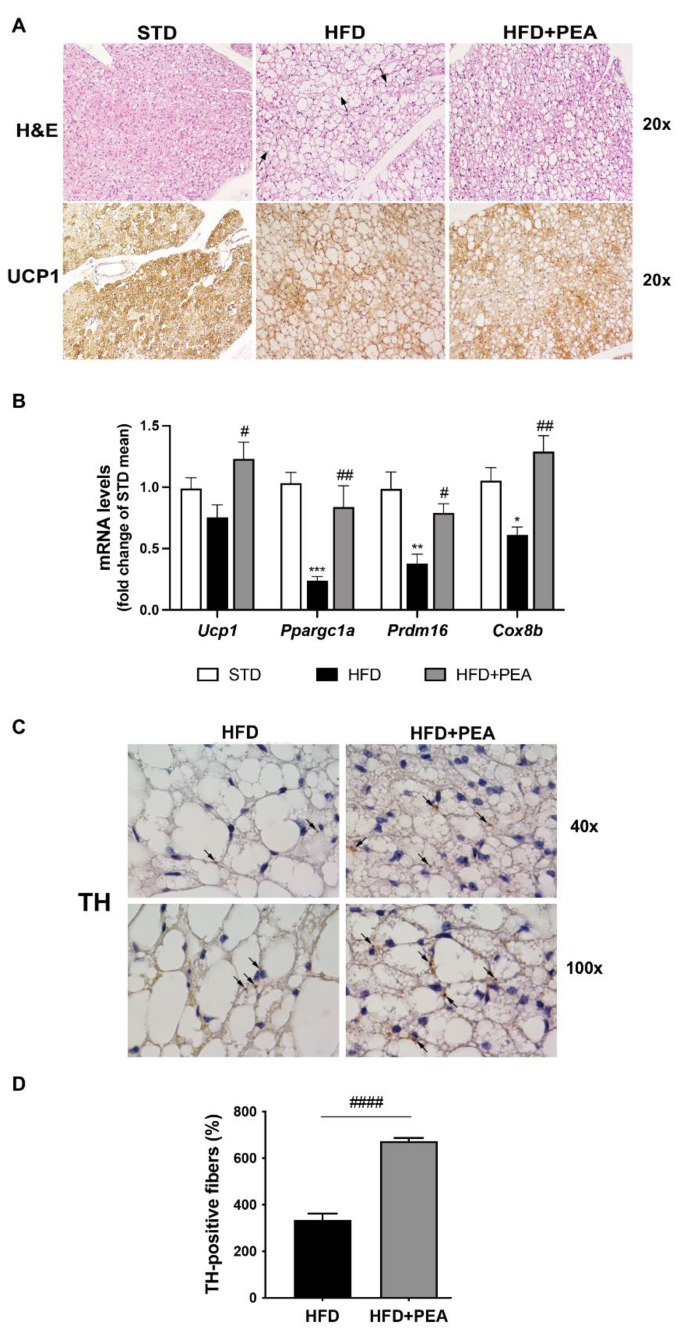
PEA recovers BAT morphology and function altered in obese mice. (**A**) Representative H & E (upper panel) and immunohistochemical staining for UCP1 (lower panel) of iBAT are reported (original magnification 20×) (n = 7 animals for each group). Paucilocular adipocytes are indicated by black arrows. (**B**) mRNA transcription levels of *Ucp1, Ppargc1a, Prdm16,* and *Cox8b* in iBAT of all experimental groups are evaluated (n = 5–6 each group). (**C**) Representative immunohistochemistry for TH in iBAT of HFD and HFD + PEA mice and (**D**) total count of positive fibers are performed in a double-blinded manner (n = 7 animals for each group) (original magnification 100×). Black arrows show positive nerves. Results are shown as mean ± SEM. * *p* < 0.05, ** *p* < 0.01, *** *p* < 0.001, significantly different from STD; # *p* < 0.05, ## *p* < 0.01, #### *p* < 0.0001 from HFD.

H & E staining for iBAT of HFD animals showed deep changes in tissue morphology. In these mice, the typical multilocularity of brown adipocytes disappeared in numerous cells or was greatly reduced, and the number of paucilocular/unilocular adipocytes increased (Figure 1A). PEA-treated obese animals exhibited a mild recovery of the brown-like phenotype, characterized by the presence of multilocular lipid droplet arrangement (Figure 1A). Moreover, the increased multilocularity observed in PEA-treated animals matched with an increased UCP1 expression at immunohistochemical staining (Figure 1A). Accordingly, PEA treatment significantly restored the mRNA level of key regulator genes of BAT-associated thermogenesis (*Ucp1, Ppargc1a, Prdm16* and *Cox8b*), as shown in Figure 1B. TH-positive parenchymal noradrenergic nerves support iBAT thermogenic function [27] and are involved in beiging in WAT depots as the inguinal subcutaneous one [28]. Morphometric data of TH immunohistochemistry serial sections showed an increased density of parenchymal noradrenergic nerves in iBAT of PEA-treated mice in comparison with HFD mice (Figure 1C,D).

### 3.2. PEA Improved Adipocyte Hypertrophy, Leptin Signaling, and Inflammatory Profile in scWAT

**Figure 2:**
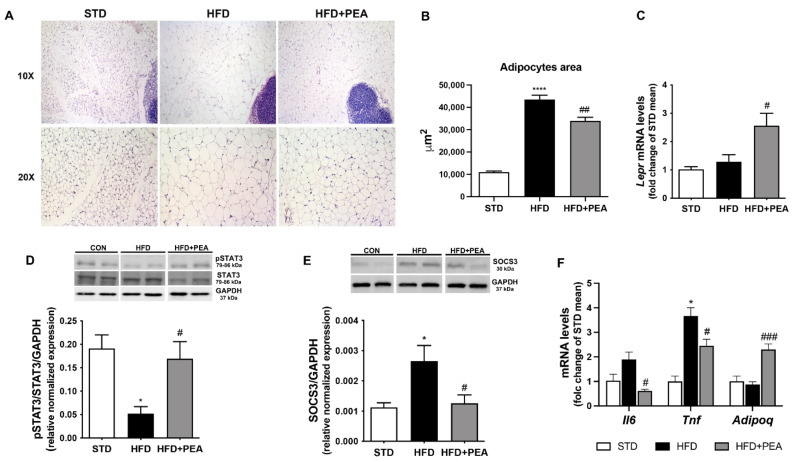
PEA promotes adipose tissue remodeling, restores leptin signaling and reduces inflammation in HFD animals. (**A**) Representative H & E of the paraffine-embedded alternate section of scWAT for all experimental groups (n = 7 animals for each group). (**B**) The mean of adipocyte size in scWAT is also shown. (**C**) Real-time PCR of leptin receptor (*LepR*) and protein expression of (**D**) phospho-STAT3 and (**E**) SOCS3 are evaluated in scWAT (n = 5–7 each group). (**F**) The mRNA transcription of *Il6*, *Tnf*, and *AdipoQ* in scWAT are measured (n = 5–6 each group). Results are shown as mean ± SEM. * *p* < 0.05, **** *p* < 0.0001 significantly different from STD; # *p* < 0.05, ## *p* < 0.01, ### *p* < 0.001 from HFD.

Light microscopy image for scWAT of HFD mice exhibited a complete loss of adipocyte multilocularity and a significant hypertrophy of adipocytes with a unilocular distribution of the lipid content (Figure 2A). PEA-treated animals showed a significant reduction of subcutaneous adipocyte size (Figure 2B). Any UCP1-positive adipocytes were detectable and parenchymal noradrenergic nerves were very rarely found in scWAT of obese mice treated or not with PEA (data not shown). Even if HFD did not reduce leptin receptor mRNAs (Figure 2C), an evident impairment of leptin signaling pathway in scWAT was observed (Figure 2D,E). Interestingly, PEA treatment increased leptin receptor mRNA transcription (Figure 2C) and restored the phosphorylation of STAT3 (Figure 2D), a downstream target of leptin receptor involved in hormone signaling. Furthermore, PEA reduced SOCS3 protein expression (Figure 2E) whose increase is involved in leptin resistance and obesity. Consistently, the inflammatory profile altered in scWAT of HFD mice was significantly restored by PEA treatment as shown by the reduction of cytokine transcription (i.e., IL-6 and TNF-α) and the increase in adiponectin (Figure 2F).

### 3.3. PEA Increased Thermogenic Markers and Induced Reprogramming of scWAT in Obese Mice

**Figure 3:**
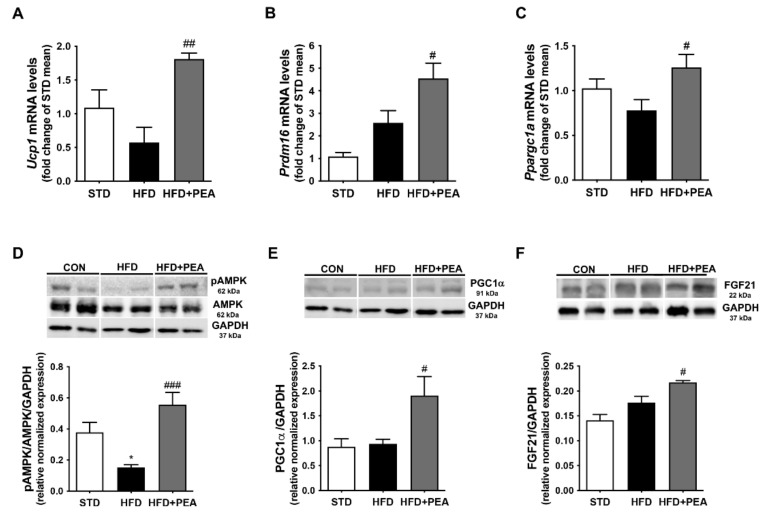
PEA induces white-to-beige tissue markers in scWAT of obese mice. mRNA transcription levels of (**A**) *Ucp1*, (**B**) *Prdm16* and (**C**) *Ppargc1a* of all groups are examined (n = 5–6). Protein expressions of (**D**) phospho-AMPK, (**E**) PGC1α and (**F**) FGF21 in scWAT of all animals are analyzed (n = 6). Results are shown as mean ± SEM. * *p* < 0.05, significantly different from STD; # *p* < 0.05, ## *p* < 0.01, ### *p* < 0.001 from HFD.

To elucidate PEA capability in modulating thermogenic mediators’ expression, we determined the mRNA level of *Ucp1, Prdm16* and *Ppargc1a* in scWAT (Figure 3A–C). PGC-1α is a critical transcriptional coactivator that induces UCP1 expression, and it is required for cellular energy regulation by AMPK via the enzyme phosphorylation [29]. Interestingly, PEA induced not only the transcription of mRNA level of all these genes (Figure 3A–C), but also the phosphorylation of AMPK that was significantly down-regulated in HFD mice, and the protein expression of PGC1α (Figure 3D,E). Moreover, PEA increased the expression of FGF21 (Figure 3F), an adipocyte secreted protein able to promote the browning effect mainly through the induction of PGC-1α [30].

### 3.4. PPAR-α Involvement in PEA-Mediated Metabolic Reprogramming of Adipocytes

**Figure 4:**
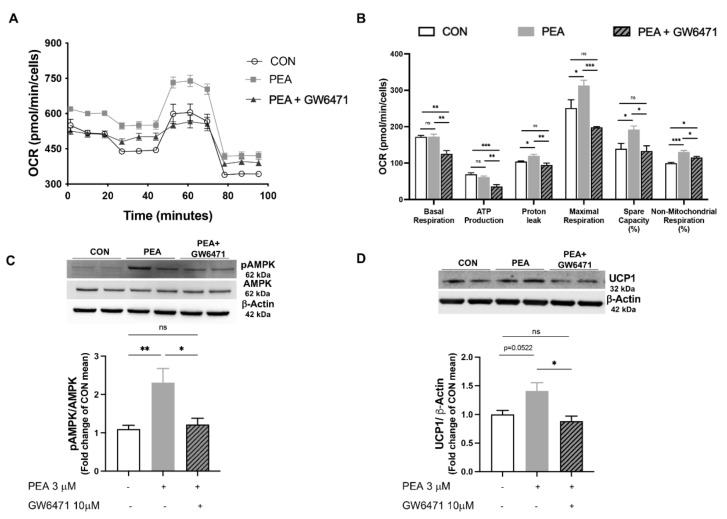
PEA exerts its metabolic effects via PPAR-α activation in differentiated 3T3-L1 cells. (**A**) Mito stress assay is performed in differentiated adipocytes, in the presence or not of PEA (3 μM) and/or the PPAR-α antagonist GW6471 (10 μM), by the Seahorse analyzer XFe24; (**B**) key parameters of mitochondrial function are reported. Western blot analysis for (**C**) phospho-AMPK and (**D**) UCP1 are also displayed. Results are shown as mean ± SEM of three different sets of experiments for 3T3-L1, respectively. * *p* < 0.05, ** *p* < 0.01, *** *p* < 0.001 significantly differ from CON.

To determine mechanistic insights by which PEA exerts its effect on adipocyte metabolic reprogramming, we analyzed mitochondrial bioenergetics of differentiated 3T3-L1 cells treated with PEA in the presence or not of GW6471, a potent PPAR-α antagonist (Figure 4A,B). PEA treatment (3 μM for 24 h) promoted OCR in differentiated 3T3-L1 (Figure 4A), mainly boosting proton leak, mitochondrial respiration in the presence of the stressor FCCP, as well as the spare capacity (Figure 4B). According to our hypothesis, GW6471 pre-treatment dampened PEA effect on mitochondrial functioning. Moreover, PEA treatment increased the phosphorylation of AMPK (Figure 4C) and UCP1 expression (Figure 4D), both effects blunted by the pre-treatment with receptor antagonist, suggesting a crucial involvement of PPAR-α activation in PEA-mediated metabolic and thermogenic activities.

### 3.5. PEA Promoted Adipogenesis in Human Adipose Stromal Cells

**Figure 5:**
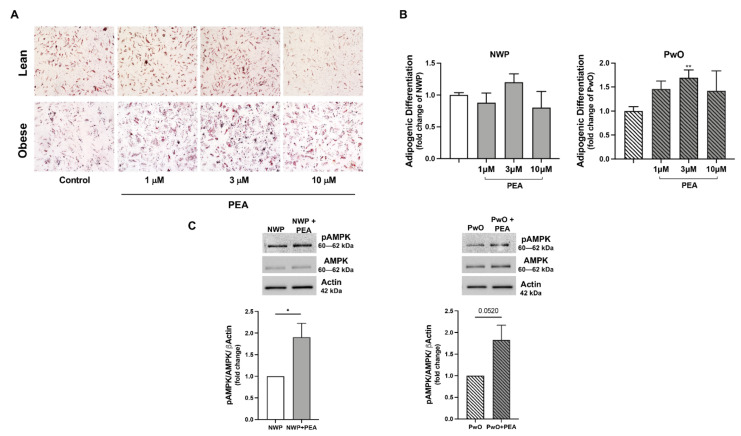
PEA induces adipogenesis in human ASCs. (**A**) Oil Red O staining of differentiated ASCs from NWP and PwO, in the presence or not of PEA (1, 3, and 10 μM) is reported. (**B**) Their degree of differentiation is also determined. (**C**) The Western blot for phospho-AMPK of cell lysates from NWP and PwO is also performed. Results are shown as mean ± SEM of three different sets of experiments for ASCs cells, respectively. * *p* < 0.05, ** *p* < 0.01, significantly differ from CON.

Finally, we verified the potential and translational effects of PEA using human ASCs, isolated from NWP or PwO, and then treated with increasing PEA concentrations (1, 3, 10 μM) in a permissive media inducing adipocyte differentiation of the human progenitor stromal fraction (Figure 5E–G). As shown in Figure 5E,F, PEA did not significantly modify the number of differentiated adipocytes of NWP, measured by Oil Red O staining; however, the in vitro PEA stimulation promoted a concentration-dependent increase in the differentiated stromal fraction from PwO reaching a significance at 3 μM (Figure 5E,F). Notably, we also found an increase in phosphorylated AMPK in both NWP and PwO groups (Figure 5G), confirming the ability of PEA (3 μM) in activating the metabolic machinery in human adipocytes.

## 4. Discussion

Basic and pharmacological research work is forward-looking to discover targets and molecules that can stimulate brown adipocytes and/or induce the browning of white adipocytes [1]. PEA, a non-canonic endocannabinoid, is recognized as an insulin sensitizer and AMPK activator, able to improve hepatic lipid and glucose homeostasis, steatosis, insulin resistance and serum leptin/adiponectin ratio [12]. Here, we study the intriguing possibility to affect adipose tissue structure and function using PEA, as a PPAR-α ligand in a murine model of obesity induced by HFD and in vitro adipocytes model. Brown adipocytes are specialized in the energy expenditure through non-shivering thermogenesis, a process that produces heat either by UCP1-dependent uncoupling of mitochondrial respiration or by other independent mechanisms [3,31]. Our data demonstrate that PEA treatment promoted the iBAT thermogenic adaptation to the excessive energy gained from the HFD, increasing the number of UCP1-positive clusters of brown adipocytes associated with the recovery of the typical BAT morphology. Although UCP1-ablated mice do not spontaneously develop obesity, they are more susceptible to high-fat feeding and prone to gain fat adiposity [32]. PPAR-α regulates both lipolytic (i.e., CPT1) and thermogenic genes (i.e., UCP1 and PGC1α) in hepatocytes and brown adipocytes [33] and PPAR-α deficient mice displayed decreased fatty acid oxidation or thermogenesis-related alterations in response to cold [34]. Specifically in BAT, the PPAR-α-mediated transcriptional regulation occurs through the cooperation with PGC1α. The PPAR-α-PGC1α complex promotes the UCP1 expression [35] and induces PRDM16, triggering a positive loop able to potentiate PGC1α expression [36]. Consistently, our data evidenced that PEA restores the expression of thermogenic genes (*Ucp1*, *Prdm16*, *Pgc1a*, *Cox8b*) reduced by HFD, and markedly increases TH-positive parenchymal nerves compared to HFD mice. Sensory nerves and metabolic hormones transpose information about energy status from adipose tissue to the brain, which in turn regulates BAT and WAT metabolism through efferent pathways. Sympathetic fibers encircle BAT vasculature and run among adipocytes; nerve-derived norepinephrine acts on brown adipocytes through adrenergic receptors to generate heat via UCP1. Indeed, denervation of BAT leads to the loss of UCP1 expression and decreased mitochondrial function in animals exposed to cold or overfeeding [37]. Among the factors secreted by the adipose tissue, leptin and adiponectin are considered real hormones, able to exert peripheral and central endocrine responses [38]. In obesity, the interplay between adipocytes and resident or circulating macrophages triggers inflammation within the adipose tissue contributing to the metainflammation [9]. Our data show that PEA exerted its anti-inflammatory properties, significantly decreasing inflammatory cytokines and increasing adiponectin in scWAT of obese mice.

Our results strongly show for the first time that PEA can promote adipose tissue plasticity not only inducing UCP1 and related thermogenic genes but also restoring leptin signaling in WAT. Leptin has an important role in the long-term regulation of body weight; increased leptin levels were found in the plasma of patients with obesity, suggesting a resistance to hormone effects on target organs when excessively produced [39]. Our previous data showed that PEA treatment reduced hyperleptinemia in obese mice and increase serum adiponectin [12]. Here, we demonstrated that PEA restored the leptin signaling pathway in scWAT, promoting the phosphorylation of the downstream target of leptin receptor STAT3. As well known, the phosphorylation of STAT3 is critical for receptor-mediated leptin signaling. Indeed, it mediates the central and peripheral effects of the hormone, and it is a useful marker of leptin sensitivity [40,41]. Previous evidence showed that the JAK/STAT pathway restoration improved leptin physiological activity in terms of body weight and metabolic control [42]. Besides its capability to lessen body weight, fat mass and energy intake in obese mice (Figure S1), PEA also reduced the expression of the inhibitory SOCS3, a member of the cytokine signaling suppressor family. Ref. [43] Beyond the onset of leptin resistance [44], the increase in SOCS3 impaired the activation of AMPK, a fuel-sensing enzyme whose activity is finely regulated by leptin in WAT [45].

The conversion of scWAT toward a healthier brown fat-like phenotype by PEA is supported by a significant increase in UCP1 expression and BAT-associated genes and transcriptional factors, including PRDM16. In particular, the expression of PRDM16 is required for scWAT conversion into beige phenotype [46], likely via a direct PPAR-α activation [47]. Recently, it has been demonstrated that PRDM-16 and PGC1α, acting as the primary regulators of mitochondrial biogenesis, may be triggered by leptin in an AMPK/STAT3-dependent manner to promote beige adipocytes in obese mice [48]. The AMPK deletion in adipocytes exacerbates insulin resistance and hepatic steatosis through impaired brown and beige fat function [17]. Our previous findings addressed PEA as an AMPK activator able to manage hepatic metabolism and mitochondrial function, integrating nutritional, energetic, and pharmacological signals [12]. Here, in mature 3T3-L1 adipocytes PEA not only significantly induced mitochondrial bioenergetics, but also increased AMPK phosphorylation and UCP1 expression. All these effects were markedly blunted by the PPAR-α antagonist GW6471. Recent findings report that adipogenesis may have a beneficial role in the metabolic homeostasis of adipose tissue, offsetting the dysmetabolism due to obesity. Therefore, the identification of mechanisms and regulators of this adaptive process is now emerging [49]. Notably, PEA stimulated the adipogenic differentiation of the human ASCs in patients with obesity. We hypothesize that the treatment with PEA enhances the recruitment of adipose progenitors since it has been demonstrated that an impaired turnover of the progenitor fraction reduces adipogenic differentiation in human hypertrophic obesity [50,51]. Additionally, PEA also increased the levels of phosphorylated AMPK suggesting the role of PEA in activating metabolic machinery even in human adipocytes. Regardless, the effects of PEA in human ASC require future investigations.

## 5. Conclusions

Our study identifies PEA and PPAR-α activation as an adipocyte-based strategy to promote the conversion of energy-storing white into energy-consuming brown-like adipocytes. The metabolic, thermogenic, and anti-inflammatory effects of PEA on adipose tissue reprogramming are the results of several converging mechanisms which may contribute to the weight and fat mass loss, the overcoming of leptin resistance and the recovery of adipose tissue homeostasis.

## Data Availability

Not applicable.

## Supplementary Materials

The following supporting information can be downloaded at: https://www.mdpi.com/article/10.3390/pharmaceutics14020338/s1, Figure S1: PEA reduced body weight, fat mass and energy intake in obese mice feeding HFD.

## Appendix A

### Appendix A.1. Animal Procedures

All animal procedures, including the experimental endpoint, were approved by the Institutional Animal Care and Use Committee (CSV) of the University of Naples Federico II under protocol no. 982/2017-PR. Body weight, food intake and animal health were weekly monitored by researchers and animal caregivers. Energy intake was calculated from food intake considering the energy content of both diets (15.8 and 21.9 kJ/g for chow diet and HFD, respectively). At the end of the experimental protocol, bioelectrical impedance analysis was performed to determine fat body composition assessment using BIA 101 analyzer, modified for the mouse (Akern, Florence, Italy). Then, mice were anaesthetized by enflurane followed by cervical dislocation and adipose tissues were collected. For morphological studies, a subset of animals was euthanized with an overdose of anesthetic (Avertin, Sigma-Aldrich, Milan, Italy) and immediately perfused with 4% paraformaldehyde (PFA) (Electron Microscopy Sciences, Hatfield, USA) in 0.1 M phosphate buffer (PB), pH 7.4.

### Appendix A.2. Morphological Studies

Interscapular BAT (iBAT) and subcutaneous WAT (scWAT) depots were dissected and further fixed by immersion in 4% PFA in PB overnight at 4 °C. The tissues were dehydrated in ethanol, cleared in xylene, and embedded in paraffin. Serial paraffin sections (3 μm thick) were obtained from each adipose depot, placed on glass slides, and dried. Alternate sections were stained with hematoxylin and eosin (H & E) to assess morphology, and for immunohistochemical procedures to evaluate tissue protein expression. Tissue sections were examined with a Nikon Eclipse E800 light microscope, and digital images were captured at 10× and 20× with a Nikon DXM 1200 camera (Nikon Instruments S.p.A, Calenzano, Italy). Adipocyte areas were measured with the open-source image analysis software ImageJ v1.46r (Rasband, WS, ImageJ; National Institutes of Health). For each experimental group, 5 fields from 5 non-overlapping H & E-stained sections per animal were analyzed.

For immunohistochemistry, 3-μm-thick paraffin-embedded sections of the fat depots were dewaxed, treated with 10% H_2_O_2_ for 5 min to block endogenous peroxidase, rinsed with PBS, and incubated in 3% normal serum blocking solution (in PBS; 30 min). Sections were then incubated overnight at 4 °C with sheep polyclonal anti-tyrosine hydroxylase (TH) antibody (dilution 1:400, Merk-Millipore, Massachusetts, United States), or rabbit polyclonal anti-UCP1, (dilution 1:1000, Abcam, Cambridge, UK). After a thorough rinse in PBS, sections were incubated in 1:200 v/v, rabbit anti-sheep (TH schedule) or goat anti-rabbit (UCP1 schedule) IgG biotinylated HRP-conjugated secondary antibody solution (Vector Laboratories, Burlingame, CA, USA) in PBS for 30 min. Histochemical reactions were performed using a Vectastain ABC kit (Vector Laboratories) and Sigma Fast 3,3′-diaminobenzidine (Sigma-Aldrich, Vienna, Austria) as a substrate. Sections were counterstained with hematoxylin, dehydrated in ethanol, and mounted in Eukitt^®^ mounting medium (Sigma-Aldrich). Staining was never observed when the primary antibody was omitted.

TH density was determined by randomly dividing the sections (n = 7 for each experimental group) in 6 fields and counting the total number of positive fibers in each section compared to the total number of adipocytes and was expressed as TH fibers number/100 adipocytes.

### Appendix A.3. Western Blot

Tissue lysates were subjected to SDS-PAGE and blots were probed with rabbit monoclonal anti-phospho 5′ AMP-activated protein kinase (AMPK) or mouse monoclonal anti-AMPK (1:1000, Cell Signaling Technology, Danvers, MA, USA), mouse monoclonal anti-PPARG coactivator 1α(PGC1α, 1:1000, Elabscience, Houston, Texas), rabbit polyclonal anti-fibroblast growth factor (FGF)21 (1:1000, Elabscience, Houston, Texas), rabbit monoclonal anti-phosphorylated signal transducer and activator of transcription 3 (pSTAT3) (1:1000, Cell Signaling Technology, Danvers MA, USA) or rabbit monoclonal STAT3 (1:500 Cell Signaling Technology, Danvers MA, USA) or rabbit polyclonal anti-suppressor of cytokine signaling 3 (SOCS3) (1:500, Santa Cruz Bio-Technology Inc., Santa Cruz, CA, USA). Western blot for GAPDH (1:8000, Sigma-Aldrich, Milan, Italy) and actin (1:1000, Sigma-Aldrich, Milan, Italy) was performed to ensure equal sample loading. Bands were detected by ChemiDoc imaging instrument (Bio-Rad Laboratories srl, Milan, Italy).

### Appendix A.4. In Vitro Experiments

3T3-L1 mouse fibroblast cells were purchased from European Collection of Animal Cell Cultures (Salisbury, Wiltshire, UK) and differentiated into adipocytes, as previously described (Ferrante MC et al., 2014). Cells were maintained in standard Dulbecco’s modified Eagle’s medium supplemented with 10% FBS and 100 U/mL penicillin and 100 μg/mL streptomycin (Gibco, Thermo Fisher Scientific, Waltham, MA, USA). Cells were incubated in adipogenesis-inducing medium, (DMEM containing 10 μM dexamethasone, 0.5 mM 3-isobutyl-1-methyl-anthine, 10 μg/mL insulin) for 2 days. Then, the medium was replaced with adipogenesis maintaining medium (DMEM containing 10 μg/mL insulin) for 2 days. Preadipocytes were kept in DMEM until day 8th from the differentiation induction. Mature adipocytes were incubated with GW6471 (10 μM). One hour later, cells were stimulated with PEA (3 μM, dissolved in EtOH) and harvested after 24 h. In another subset of experiment, undifferentiated 3T3-L1 cells were seeded in XFe24 cell culture plates at 1 × 103 density. After differentiation, cells were treated with the tested compounds, as described above. Before starting the assay, the medium was replaced with Seahorse base medium (supplemented with 1 mM pyruvate, 10 mM glucose and 2 mM glutamine, pH 7.4) and the cells were incubated at 37 °C in a non-CO_2_ incubator for 1 h. The oxygen consumption rate (OCR) was measured under a basal or stressed condition by sequentially adding 1 μM of oligomycin, 0.75 μM of FCCP and 1 μM of rotenone/antimycin A to measure all the key parameters of mitochondrial respiration, including basal respiration, ATP production, proton leak, maximal respiration, spare capacity, and non-mitochondrial respiration. The value of OCR was normalized to cell number.

ASCs were seeded at a density of 4000 cells/cm^2^ in DMEM-Low glucose in 10% fetal bovine serum (FBS)/200 mM L-glutammine/100 mM sodium pyruvate/non-essential aminoacids/penicillin/amphotericin. At passage 3, ASC cultures were subjected to adipogenic differentiation for 21 days by seeding 1 × 104 cells/cm^2^ cells in StemPro^®^ adipogenesis differentiation kit (GIBCO, Grand Island, NY, USA) in the presence or not of PEA (1, 3, 10 μM). Accumulation of lipid droplets was evaluated by Oil Red O staining (Sigma-Aldrich, St. Louis, MO, USA). Both the medium and PEA were refreshed every 3–5 days. Adipogenesis was quantified in 10 randomly selected fields observed under an optical microscope. The adipogenic differentiation was calculated as a ratio of positive adipogenic cells number/total cells number and then normalized on control cells (ASCs without PEA in presence of adipogenic differentiation medium).

### Appendix A.5

**Table A1:** Primers for Real-Time PCR.

Genes (Qiagen)	Catalog N°	RefSeq Transcript
*Ucp1*	22227	NM_009463
*Ppargc1a*	19017	NM_008904
*Prdm16*	70673	NM_001177995
*Cox8b*	12869	NM_007751
*Il6*	16193	NM_031168
*Tnf*	21926	NM_013693
*Adipoq*	11450	NM_009605
*Lepr*	249900	NM_146146
*Actb*	11461	NM_007393
*Rn18S*	19791	NR_003278
