# Supplementary material for: Palmitoylethanolamide Promotes White-to-Beige Conversion and Metabolic Reprogramming of Adipocytes: Contribution of PPAR-α
Source: Pharmaceutics. 2022 Jan 31;14(2):338. doi: 10.3390/pharmaceutics14020338 (PMC8880285; doi:10.3390/pharmaceutics14020338)
Supplement: Supplementary file 1 [file pharmaceutics-14-00338-s001.zip › pharmaceutics-1542535-supplementary.pdf]

pharmaceutics

MDPI

# Supplementary Materials: Palmitoylethanolamide Promotes White-to-Beige Conversion and Metabolic Reprogramming of Adipocytes: Contribution of PPAR-α

**Chiara Annunziata, Claudio Pirozzi, Adriano Lama, Martina Senzacqua, Federica Comella, Antonella Bordin, Anna Monnolo, Alessandra Pelagalli, Maria Carmela Ferrante, Maria Pina Mollica, Angelo Iossa, Elena De Falco, Giuseppina Mattace Raso, Saverio Cinti, Antonio Giordano and Rosaria Meli**

*PEA reduced body weight, fat mass and energy intake in obese mice feeding HFD*

The experimental protocol is reported in Figure S1a. Body weight gain of all groups (n = 10 each group) is reported in Figure S1b, starting from 0 to 19th week. PEA induced modifications in body weight gain reaching the significance after 5 weeks of treatment (at 18th and 19th week). Accordingly, PEA treatment reduced fat mass (Figure S1c). PEA did not change the energy intake weekly measured, but interestingly the area under the curve (AUC) values showed a significant reduction of total energy intake induced by PEA during 7-weeks treatment (Figure S1d).

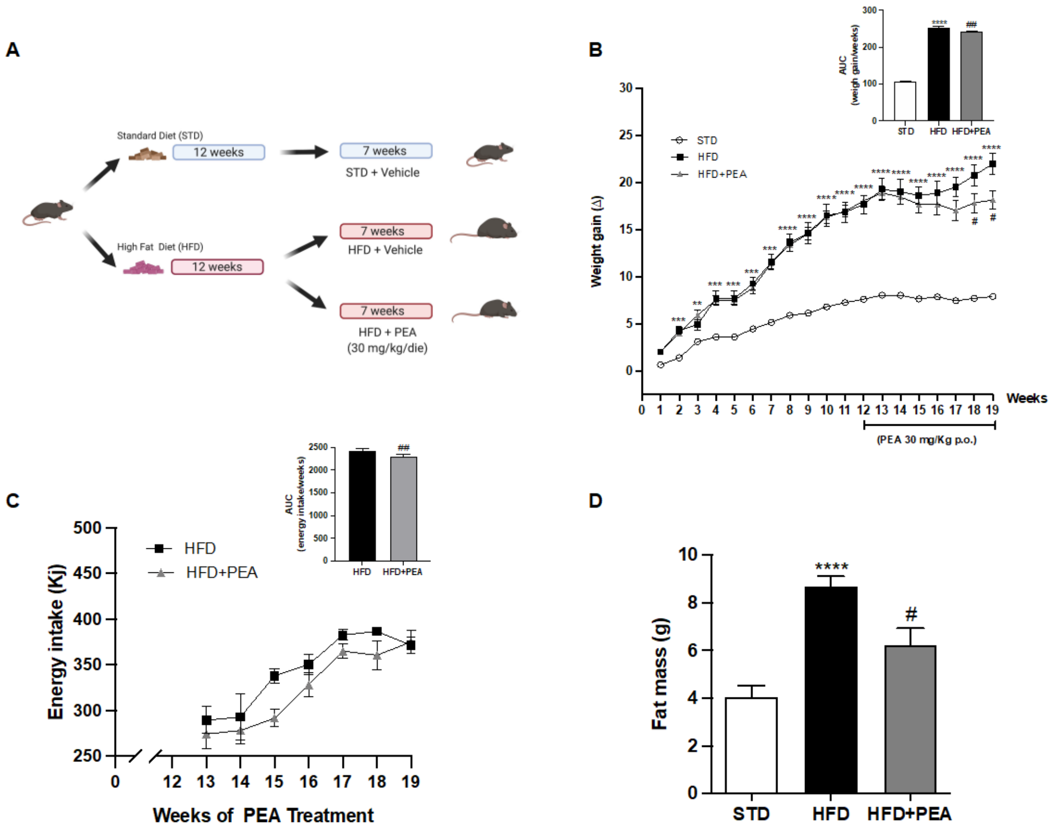


**Figure S1.** PEA treatment ameliorates metabolic and endocrine parameters in HFD mice. (**A**) In vivo experimental protocol. (**B**) The effects of PEA treatment on body weight gain, (**C**) energy intake measured throughout the experimental period (0-7 weeks of treatment after 12-week HFD feeding) and (**D**) fat mass, and (n = 10 each group) are reported. Data are presented as means ± SEM. $**P < 0.01$, $***P < 0.001$, $****P < 0.0001$ significantly different from STD; #P<0.05, ##P<0.01, from HFD.
